# Insight into the Diversity of Penicillin-Binding Protein 2x Alleles and Mutations in Viridans Streptococci

**DOI:** 10.1128/AAC.02646-16

**Published:** 2017-04-24

**Authors:** Mark van der Linden, Julia Otten, Carina Bergmann, Cristina Latorre, Josefina Liñares, Regine Hakenbeck

**Affiliations:** a German National Reference Center for Streptococci, Department of Medical Microbiology, University Hospital RWTH Aachen, Aachen, Germany; b Department of Microbiology, University of Kaiserslautern, Kaiserslautern, Germany; c Novo Nordisk Pharma GmbH, Mainz, Germany; d Department of Microbiology, Hospital Sant Joan de Déu, University of Barcelona, Barcelona, Spain; e Microbiology Department, Hospital Universitario de Bellvitge-IDIBELL, Ciber de Enfermedades Respiratorias, University of Barcelona, Barcelona, Spain

**Keywords:** S. pseudopneumoniae, PBP2x, penicillin resistance, PBP2x mutation, viridans streptococci

## Abstract

The identification of commensal streptococci species is an everlasting problem due to their ability to genetically transform. A new challenge in this respect is the recent description of Streptococcus pseudopneumoniae as a new species, which was distinguished from closely related pathogenic S. pneumoniae and commensal S. mitis by a variety of physiological and molecular biological tests. Forty-one atypical S. pneumoniae isolates have been collected at the German National Reference Center for Streptococci (GNRCS). Multilocus sequence typing (MLST) confirmed 35 isolates as the species S. pseudopneumoniae. A comparison with the *pbp2x* sequences from 120 commensal streptococci isolated from different continents revealed that *pbp2x* is distinct among penicillin-susceptible S. pseudopneumoniae isolates. Four penicillin-binding protein x (PBPx) alleles of penicillin-sensitive S. mitis account for most of the diverse sequence blocks in resistant S. pseudopneumoniae, S. pneumoniae, and S. mitis, and S. infantis and S. oralis sequences were found in S. pneumoniae from Japan. PBP2x genes of the family of mosaic genes related to *pbp2x* in the S. pneumoniae clone Spain^23F^-1 were observed in S. oralis and S. infantis as well, confirming its global distribution. Thirty-eight sites were altered within the PBP2x transpeptidase domains of penicillin-resistant strains, excluding another 37 sites present in the reference genes of sensitive strains. Specific mutational patterns were detected depending on the parental sequence blocks, in agreement with distinct mutational pathways during the development of beta-lactam resistance. The majority of the mutations clustered around the active site, whereas others are likely to affect stability or interactions with the C-terminal domain or partner proteins.

## INTRODUCTION

Streptococcus pneumoniae is one of the major human pathogens. It colonizes the upper respiratory tract asymptomatically (1), but can lead to a variety of diseases ranging from sinusitis, otitis media, and pneumonia to meningitis (2). By contrast, related streptococci from the mitis group of viridans streptococci (SMG) are commensal organisms that rarely cause disease. S. pneumoniae is differentiated from other species by a variety of phenotypic and genotypic tests on the basis of the presence of important virulence factors carried by almost all S. pneumoniae strains (for a review, see reference 3). This includes the capsule biosynthesis cluster responsible for the expression of more than 90 serotypes (4). Moreover, S. pneumoniae cells lyse in the presence of sodium deoxycholate (DOC) due to the presence of the l-alanine amidase LytA, an autolysin that is also responsible for stationary-phase autolysis. The gene *lytA* is located on an island together with *ply* encoding a potent cytolysin (5). Furthermore, S. pneumoniae is optochin susceptible. Nevertheless, tests for these phenotypes are not completely reliable for identifying S. pneumoniae due to variations in their specificity and sensitivity (6).

S. pneumoniae can be genetically differentiated from its close relatives S. mitis and S. oralis by comparing DNA sequences of housekeeping genes, such as with multilocus sequence typing (MLST) (7), which has been considered the gold standard for studying the epidemiology of pathogenic bacteria. A greater number of species can be resolved by multilocus sequence analysis (MLSA) (8). However, several studies revealed the problems associated with identifying the species of SMG due to their competence for genetic transformation. Lateral intra- and interspecies gene transfer results in mosaic genes, a highly variable and relatively large accessory genome, and consequently, to diffuse species borders, resulting in “fuzzy species” (8). This is specifically evident in the lineages comprising S. mitis and S. oralis, thereby challenging the definition of species (6, 9–11).

The recently described S. pseudopneumoniae (12) provides additional complications for identifying SMG species. Although closely related to S. pneumoniae, S. pseudopneumoniae may be differentiated by a variety of phenotypic and molecular assays, consistent with the previous classification as “atypical S. pneumoniae.” Some of the key characteristics of S. pseudopneumoniae are the expression of optochin resistance in the presence of 5% CO_2_, the lack of a pneumococcal capsule, and bile insolubility (12). An analysis of the first S. pseudopneumoniae genome of strain IS7493 documented the lack of some pneumococcal virulence factors, such as the capsule biosynthesis genes and the choline-binding proteins PspC, PcpA, and PspA (13). However, it contained the *ply-lytA* islet. Comparative genomic hybridization showed that this islet is common among S. pseudopneumoniae strains (13) and among eight sequence types of SMG that have been identified (14, 15). The sequence of *lytA* is S. pneumoniae is distinct from that of S. pseudopneumoniae and other members of the viridans streptococci (14, 15) and can also be used to differentiate S. pseudopneumoniae by specifically designed PCR.

S. pseudopneumoniae has been isolated mainly from patients with respiratory diseases (16–19). Many of these isolates showed resistance to antibiotics, including a reduced susceptibility to penicillin. The penicillin-binding proteins (PBPs) of S. pseudopneumoniae IS7493 are more similar to PBPs of resistant S. pneumoniae than to the highly conserved PBPs of sensitive strains (13). Since this strain has a decreased susceptibility to beta-lactams, its PBPs might not be representative for this species. The spread of penicillin resistance among populations of S. pneumoniae has been observed for many decades (20), fostered by genetic transformations mediating interspecies gene transfer among closely related streptococci of the mitis group. This results in highly variable mosaic genes encoding the main players in the development of penicillin resistance, the PBPs 2x, 2b, and 1a (for a review, see reference 21). By contrast, PBPs of penicillin-sensitive S. pneumoniae are strictly conserved. Interestingly, a variety of different alleles circulate within the population of S. mitis, and some have been shown to be highly related to mosaic blocks in PBPs from penicillin-resistant S. pneumoniae (PRSP). S. mitis has been identified as the main donor of PBP sequences in PRSP (22–25).

A large number of atypical S. pneumoniae suspected to be S. pseudopneumoniae have been collected over the years at the German National Reference Center for Streptococci (GNRCS) in Germany. One goal of this study was to clarify their species identification by multilocus sequence typing (MLST). Moreover, the mosaic structures of the PBP2x genes were compared with those from a large number of sensitive and resistant commensal streptococci to reveal their relationships to *pbp2x* in S. pneumoniae and S. mitis, the closest relatives. This gene was chosen as alterations in PBP2x are essential for penicillin resistance, and thus represent a driving force for the evolution of this phenotype. Clusters of related *pbp2x* sequences were analyzed to reveal group-specific alterations associated with beta-lactam resistance.

## RESULTS

### Differentiation of S. pseudopneumoniae by MLST.

Within the large collection of invasive S. pneumoniae strains held at the German National Reference Center for Streptococci (GNRCS), 41 atypical isolates were identified that did not react with specific pneumococcal anti-polysaccharide antibodies or showed rough colony morphologies. These isolates were not consistently bile soluble, and optochin resistance was frequently expressed in the presence of CO_2_. Moreover, most of them were DOC insoluble and optochin resistant (see Table S1A in the supplemental material). A restriction analysis of PCR-amplified *lytA* with *Ppu*21I and 16SrRNA (see Materials and Methods) resulted in fragments atypical for S. pneumoniae in most cases (15). These data suggested that the strains represent S. pseudopneumoniae.

Strains that differed from S. pneumoniae according to the criteria listed above were further subjected to multilocus sequence typing (MLST) (7) to reveal their phylogenetic relationships to S. pneumoniae and S. mitis. At least five of the seven genes defined by MLST were amplified from 41 strains (see Table S1B). Genes identified by MLST of S. pseudopneumoniae IS7493, whose genome sequence is available (13), were included in further analyses. In addition, we investigated the 38 whole-genome shotgun contigs of S. pseudopneumoniae listed in GenBank. Before extracting the seven genes from MLST, we used MLSA to compare *map* and *pyk*, which differentiate S. pseudopneumoniae from S. mitis (8). Moreover, we compared the d,d-carboxypeptidase PBP3 gene, which is also remarkably species specific (10) (see Fig. S1). Only nine out of the 38 S. pseudopneumoniae genomes showed 99 to 100% identities for the MLSA loci and >94% identity for the PBP3 gene (only strain 276-03 showed a low identity for *pyk* [93%]) and were considered to be S. pseudopneumoniae. Nineteen genomes were defined as S. mitis and five each were defined as S. infantis and S. oralis (see Table S1C).

For the final MLST analysis, all of the S. pseudopneumoniae and S. mitis were included and compared with MLST data from 29 S. pneumoniae and 36 S. mitis strains from different parts of the world (22); S. oralis Uo5 (26) was used as a reference for this species. Concatenated sequences of six genes were analyzed by the MEGA6 program, including those from 23 atypical strains of this study. The gene *ddl* was omitted as it is frequently highly diverse in penicillin-resistant isolates. The reason is that it maps closely to *pbp2b*, which was acquired by horizontal gene transfer during the evolution of penicillin resistance (27).

**FIG 1:**
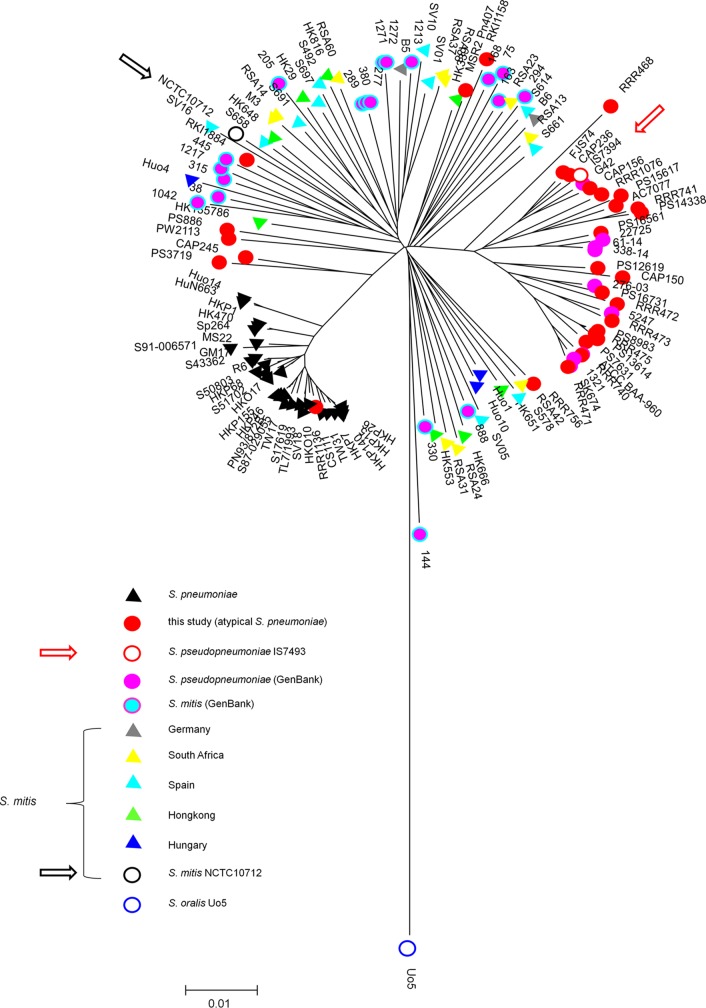
MLST tree of strains from this study and reference S. pneumoniae and S. mitis strain. A neighbor-joining tree was constructed using the concatenated sequences of six MLST loci, excluding *ddl* from 30 atypical S. pneumoniae strains from this study, combined with reference S. mitis and S. pneumoniae from Chi et al. (22). In addition, MLST genes were extracted from the genomes of S. pseudopneumoniae IS7394, 9 S. pseudopneumoniae, and 19 S. mitis listed in GenBank under whole-genome shotgun contigs of S. pseudopneumoniae (see Table S1B in the supplemental material). S. oralis Uo5 was included as a reference for this species. Bootstrap values (percentages) are based on 1,000 replications. The bar refers to genetic divergence as calculated by the MEGA software.

The phylogenetic tree clearly differentiated between S. pneumoniae, S. mitis, and the sole S. oralis strain Uo5 (Fig. 1). In addition, one large cluster of S. pseudopneumoniae strains was observed that included 21 of the atypical isolates, strain IS7394, and all nine strains identified as S. pseudopneumoniae from GenBank. Four atypical isolates were located on two branches between S. mitis and S. pneumoniae (PS3719, CAP245, PS2113, and PS886). All 19 strains from GenBank identified as S. mitis as described above clustered within the S. mitis group, as did the four atypical strains RKI1158, RKI884, RRR756, and MSR2_Pn407. RRR1136 was found among S. pneumoniae (Fig. 1). This strain was shown by a later analysis to express a rare serotype 31, which was not identified in the serotype analysis, and thus had been classified initially as a nontypeable strain. The MLST genes either matched known alleles of S. pneumoniae or differed from known alleles in up to 4.6%; only RRR468 contained one allele (*recP*) that is common among S. oralis.

The seven MLST loci were also analyzed individually (see Fig. S2 in the supplemental material). The phylogenetic tree generated with *aroE* sequences showed a tight cluster of 40 strains well separated from other branches, and also, the tree generated with *recP* showed one cluster of 30 S. pseudopneumoniae strains that included two S. mitis (Fig. S2). Smaller clusters of 8 to 26 strains that consisted only of S. pseudopneumoniae were obtained with the other six alleles, and one S. mitis was found within the *spi* cluster of 12 S. pseudopneumoniae strains (Fig. S2). Clustering of S. pseudopneumoniae alleles within S. pneumoniae was also observed.

Forty-two strains (32 atypical strains and the 10 sequenced S. pseudopneumoniae strains) contained at least four of the six MLST alleles in S. pseudopneumoniae/S. pneumoniae clusters. Strains PS3719 and RRR468 contained two MLST alleles, and strains PS886 and PW2113 contained only one (*aroE* and/or *recP*) positioned within S. pseudopneumoniae/S. pneumoniae clusters (Table S1B), but they still clustered with S. pseudopneumoniae in the MLST analysis that included six alleles (Fig. 1). Further analyses are required to confirm the species of these strains. Finally, the MLST data identified RKI1158, RKI884, RRR756, MSR2_Pn407, and RRR720 as S. mitis, as were the 19 strains from GenBank (Table S1A and S1B).

### Phylogenetic analysis of PBP2x.

One purpose of this part of the study was to see how divergent PBP2x genes are in S. pseudopneumoniae. Moreover, the diversity of *pbp2x* in S. mitis was analyzed to further our understanding on the distribution of such sequences among S. pseudopneumoniae and other SMG and to identify mutations relevant for developing resistance. Sequence information covered all three PBP2x domains to gain more insight into gene transfer events. Unique penicillin-sensitive *pbp2x* alleles for SMG (MIC for penicillin G, <0.05; for cefotaxime, <0.003), not containing any mutations known to be relevant for penicillin resistance, were used as reference sequences for defining mosaic blocks. These include *pbp2x* in S. pneumoniae R6 and S. mitis M3, and in NCTC10712, which contains large unique sequence blocks predominantly within the transpeptidase domain and flanked by S. mitis M3-related sequences as, described recently (23), as well as two unique *pbp2x* alleles in S. mitis 658 and SV01 from the Kaiserslautern strain collection. Finally, *pbp2x* in the PRSP clone Spain^23F^-1 was used as a reference for resistant mosaic genes, as it is widespread among penicillin-resistant SMG (22); it contains sequences related to *pbp2x*_M3_ mainly in the transpeptidase domain.

In addition to *pbp2x* of S. pseudopneumoniae, we analyzed 38 genes from GenBank listed in Table S1C in S. mitis and S. oralis from our collection, including nonhuman isolates (10). Recently, eight clusters of *pbp2x* in SMG have been described on the basis of sequences covering the transpeptidase domain (25). We included *pbp2x* sequences representative of three of these clusters that did not match any of the reference *pbp2x* sequences described above, as well as singletons from this study. Furthermore, we searched GenBank for S. pneumoniae *pbp2x* that contained blocks of the reference sequences.

**FIG 2:**
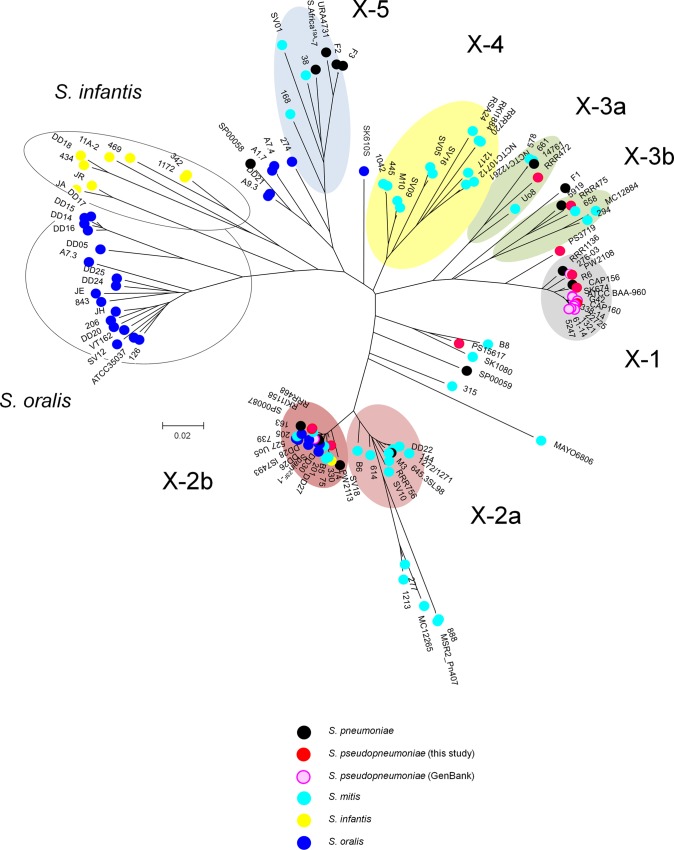
Comparative analysis of PBP2x genes. An evolutionary tree was generated from 95 PBP2x sequences using the MEGA software. Clusters X-1 to X-5 were defined according to the reference sequences of *pbp2x* in S. pneumoniae R6 (X-1) and those of S. mitis strains M3 (X-2b), 658 (X-3a and 3b), NCTC10712 (X-4), and SV01 (X-5). S. oralis and S. infantis *pbp2x* clusters are also indicated. Cluster X-2b refers to the family of mosaic genes related to *pbp2x* in the S. pneumoniae clone Spain^23F^-1. The species are indicated by different colors. Bootstrap values (percentages) are based on 1,000 replications. The bar refers to genetic divergence as calculated by the MEGA software.

A total of 120 *pbp2x* sequences comprising 104 distinct alleles were phylogenetically analyzed using the MEGA6 program. Five clusters were defined that contained one reference sequence each (Fig. 2): S. pneumoniae R6 (cluster X-1) and S. mitis strains M3 (X-2a), 658 (X-3a), NCTC10712 (X-4), and SV01 (X-5). Sequences of *pbp2x* related to the mosaic *pbp2x* of the PRSP clone Spain^23F^-1 were found in cluster X-2b close to X-2a and consisted exclusively of genes in penicillin-resistant strains, as were those in cluster X-3b, which was close to X-3a. Fourteen *pbp2x* alleles were located on branches outside these clusters. Most *pbp2x* alleles in S. oralis formed one group, which included strain ATCC 35037, as did most of the *pbp2x* alleles of S. infantis; *pbp2x* alleles in penicillin-resistant strains of these species were found outside clusters or were part of cluster X-2b. In summary, several highly divergent *pbp2x* alleles are apparent in the three species S. mitis, S. infantis, and S. oralis.

### Mosaic structures of *pbp2x*.

**FIG 3:**
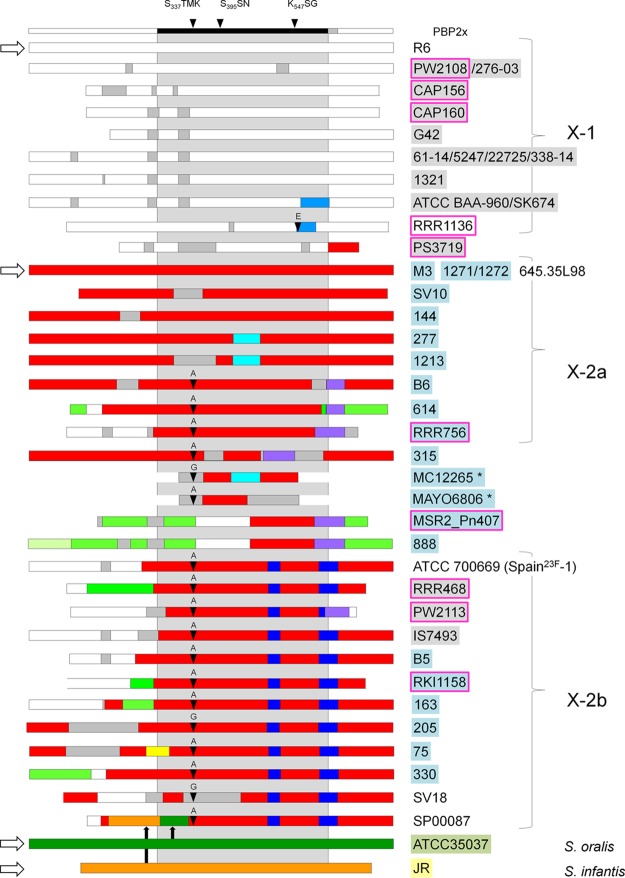

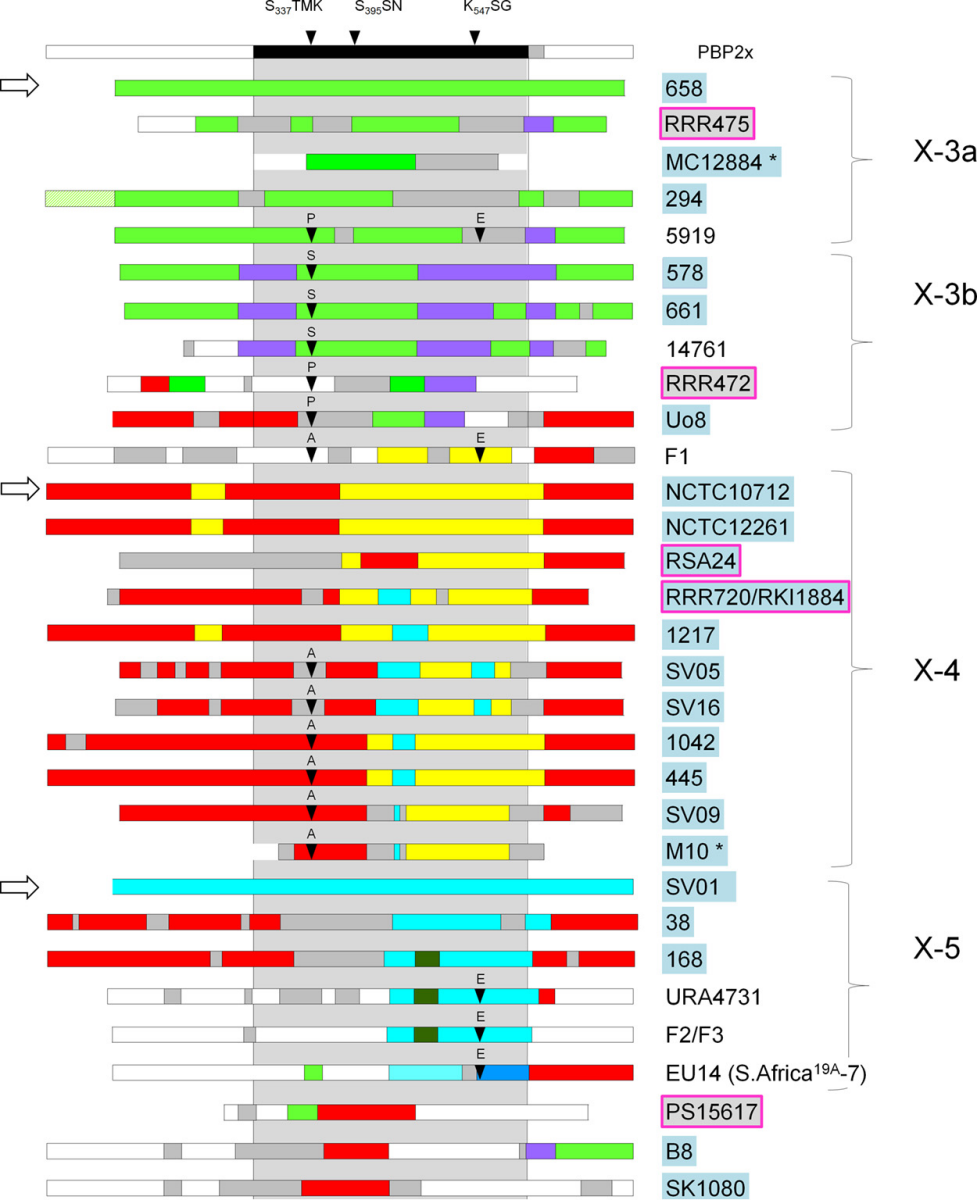
Mosaic structure of PBP2x variants. Mosaic gene structures were deduced by comparing the reference PBP2x sequences (white arrows on the left side) in S. pneumoniae R6 (white sequence blocks), S. mitis strains M3 (red), NCTC10712 (yellow), SV01 (light blue), and 658 (green), S. oralis ATCC 35037 (dark green), and S. infantis JR (orange). Highly similar sequences (<5% difference) are shown in the same colors; unrelated sequences of unknown origin are in gray. The domain structure and active site boxes of PBP2x are indicated on top; the gray shaded area indicates the central transpeptidase domain. Mutations at sites 338 and 552 are indicated by black arrows. The strains are indicated on the right and are shaded according to species: white, S. pneumoniae; light blue, S. mitis; gray, S. pseudopneumoniae; green, S. oralis; orange, S. infantis. A pink frame indicates isolates obtained from the GNRCS; four *pbp2x* from Jensen et al. (25) are indicated by an *.

**FIG 4:**
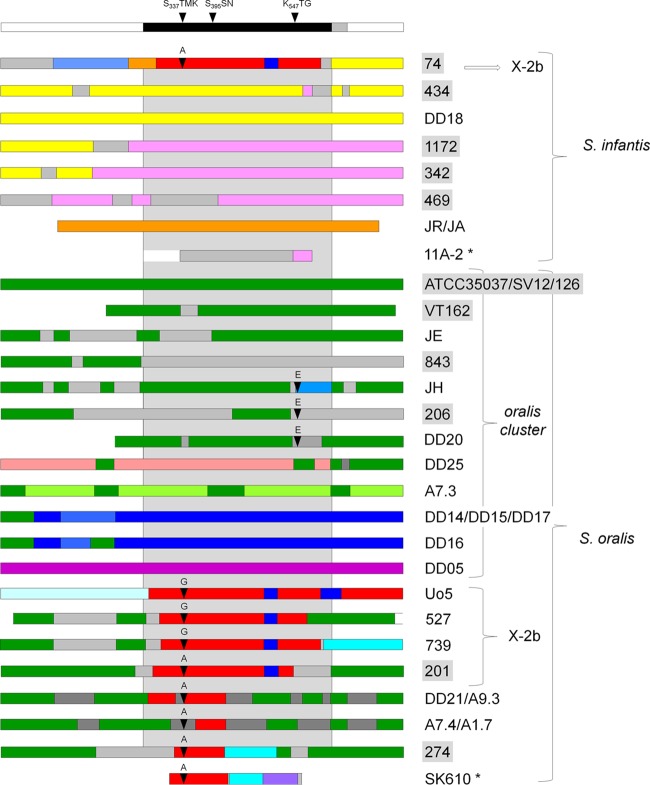
Mosaic structure of PBP2x variants of S. infantis and S. oralis. Mosaic gene structures were deduced by comparing PBP2x sequences. Highly similar sequences (<5% difference) are shown in the same colors; unrelated sequences of unknown origin are in gray. The red block in S. infantis 74 indicates the 2x-23F group. The domain structure and active site boxes are indicated on top; the central transpeptidase domain is indicated by the gray area. Mutations at sites 338 and 552 are indicated. The strains are indicated on the right. Sequences were retrieved from GenBank (gray); those described by Jensen et al. (10) are marked with *. All others have been described in (10), or are part of this study. Accession numbers: 527, KY292529; 739, KY292532; SV12, KY292537; JA, KY292545; JE, KY292547; and JH, KY292546.

The mosaic structures of the *pbp2x* alleles of S. pseudopneumoniae in comparison to those of S. mitis and S. pneumoniae are shown in Fig. 3. S. oralis and S. infantis pbp2x are shown in Fig. 4; only those of S. infantis strain JR and S. oralis strain ATCC 35037 are included in Fig. 3, as closely related sequence blocks were detected in S. pneumoniae. The overall view shows several highly divergent *pbp2x* alleles not only in S. mitis, but in S. infantis and S. oralis as well. Within one cluster, there were mainly sequences of the reference *pbp2x* in addition to sequence blocks of an unknown origin (gray in Fig. 3 and 4). Small sequence blocks of the reference *pbp2x* in S. mitis occurred in several clusters. The 14 *pbp2x* alleles which were scattered outside the clusters had unique mosaic structures and contained sequences from at least two different sources.

In the four groups related to the S. mitis reference *pbp2x*, multiple recombinations were observed that were frequently located within the central transpeptidase/penicillin binding domain (PBD), whereas the 5′- and 3′-sequences encoding the N- and C-terminal domains were more uniform. It is also remarkable that in many cases, one distinct sequence block covered the spacer region between the transpeptidase and the C-terminal domain, mimicking the structural organization of the protein. Although most mosaic blocks covered large regions of up to 450 codons, very small regions of divergence of <10 codons were noticeable. S. pneumoniae sequences were frequently observed in *pbp2x* in S. mitis.

### S. pseudopneumoniae pbp2x.

Cluster X-1 consisted almost exclusively of 12 S. pseudopneumoniae genes (7 alleles). The four sequences in strains 61-14, 5247, 22725, and 338-14 were identical, as were the *pbp2x* in strains PW2108 and 276-03 (Fig. 3). All of these contained small regions of up to 59 codons in length that differed by 7 to 20% from the R6 *pbp2x*; none of the S. pseudopneumoniae alleles were found among S. pneumoniae or S. mitis in a nucleotide BLAST search.

All of the other *pbp2x* alleles in S. pseudopneumoniae were from penicillin-nonsusceptible strains and displayed a variety of mosaic structures. They were found in clusters X-3a/b (RRR475 and RRR472) and X-2b (PW2113, RRR468, and IS7394) or did not cluster (PS3719 and PS15617). Sequences from S. mitis NCTC10712, S. oralis, or S. infantis were not found in these alleles.

### S. mitis pbp2x.

Mosaic S. mitis PBP2x variants were found in all of the clusters except X-1 (Fig. 2 and 3). In general, the individual makeup of mosaic patterns in S. mitis is remarkable, not only in penicillin-resistant isolates, but in penicillin-susceptible isolates as well. There were only a few cases of near sequence identity (>2%): *pbp2x* in S. mitis strains 1271, 1272, and DD22 differed from *pbp2x*_M3_ by ≤15 nucleotides (nt) (0.7%), and *pbp2x* in strain NCTC12261 differed from that of NCTC10712 by 45 nt (2%). Even in cases where the overall mosaic structures were very similar, individual *pbp2x* alleles differed from each other in the lengths of related blocks and the presence of distinct sequences. Examples of this are *pbp2x* in strains 1042, 445, and 1217 (X-4), 578 and 661 (X-3), and MSR2_Pn407 and 888. SV01-related sequences were found in another 14 S. mitis strains (cluster X-5, X-4, and X-2a). No sequence blocks related to S. oralis ATCC 35037 or S. infantis JR were detected. The diversity of 3′ sequences among the 23F-family (X-2b) is of interest, signifying multiple recombination events that aggravate tracking of their evolutionary history.

### S. pneumoniae pbp2x.

Sequences from each of the reference *pbp2x* alleles were detected in individual S. pneumoniae mosaic PBP2x genes, all of which were from penicillin-resistant isolates (Fig. 3). S. pneumoniae sequences related to *pbp2*x in S. mitis NCTC10712 (X-4) have been described in serotype 19A S. pneumoniae from Hungary (28) and were not included in the present analysis. In most cases, the mosaic structures covered major parts of the PBP2x genes. However, there were two exceptions: *pbp2x* of strain 645.35L98 designated S. pneumoniae (29) was completely identical to that of S. mitis M3; this is so odd that the species identification is questionable. Moreover, S. pneumoniae RRR1136 (group 1) contained only two small sequence blocks of 28 and 124 nt compared with that of *pbp2x*_R6_, an unusual mosaic structure among resistant S. pneumoniae where mosaic blocks are generally much longer. SV18 (X-2b) contained a large divergent block of unknown origin covering the region encoding the active site S_337_ (see below). PBP2x of S. pneumoniae strains 5919 and 14761 were located in group 5 (X-3), that of strain F1 was in X-4, and four strains contained sequence blocks of S. mitis SV01 (X-5) in the 3′-part of the transpeptidase domain (S. pneumoniae strain URA4731 from Portugal, F2 and F3 from France, and EU14, member of the clone S.Africa^19A^-7). S. pneumoniae from Japan contained S. oralis ATCC 35037 sequences (strains SP00058 and SP00087), and SP00087 (X-2b) contained S. infantis JR *pbp2x* sequences, as well as the regions encoding the N- and C-terminal domains. Taken together, all of the S. pneumoniae mosaic PBP2x genes showed signs of multiple gene transfer events involving S. mitis, S. infantis, and S. oralis sequences.

### Altered sites in PBP2x.

PBP2x is a 750-amino acid (aa) multidomain protein with a short N-terminal membrane anchor, an N-terminal domain, and a central transpeptidase domain (residues 266 to 626) followed by linker regions and a C-terminal extension (residues 635 to 750) folded into two PASTA domains (30).

Mutations associated with resistance have only been described within the central transpeptidase domain of the protein, which contains three boxes highly conserved in penicillin-sensitive streptococci: S_337_TMK with the active site serine, S_394_SN, and K_547_TG. PBP2x of the sensitive reference strains S. mitis M3, NCTC10712, 658, SV01, S. oralis ATCC 35037, and S. infantis JR that represent donors for mosaic blocks in PBP2x of PRSP differ from the S. pneumoniae R6 PBP2x at 215 sites (28.7%), 66 of which are within the transpeptidase domain. This number includes 23 sites listed in a recent study as being associated with resistance (31), and T_369_ mentioned by Jensen et al. (25) was present in the penicillin-susceptible S. mitis 658. These mutations were not considered in the following analysis.

The PBP2x alleles were divided into two groups: class 1, which contained at least one of the two mutations at sites 338 and/or 552 known to confer resistance to penicillin and/or cefotaxime, and class 2, which did not. Residues 338 and 552 are near the active site S_337_ and the K_547_TG box, respectively, and lead to decreased susceptibility to beta-lactams when introduced in penicillin-sensitive S. pneumoniae (32, 33). Not counting alterations that occurred in the reference PBP2x, the number of potentially interesting sites in class 1 PBP2x was reduced to 63 sites: 21 were located within the N-terminal region (265 aa), 38 within the transpeptidase domain (351 aa), and 14 in the C-terminus, including the linker region (134 aa). The numbers of sites that were altered in class 2 PBP2x only were eight in the N-terminal domain, 18 in the transpeptidase domain, and five in the C-terminal extension (see Table S2).

### Mutations at sites T_338_ and Q_552_.

Thirty-two PBP2x alleles contained mutations at T_338_ to A_338_ (21 strains), G_338_ (S. pneumoniae SV18 and S. mitis 205), P_338_ (S. pneumoniae 5919, S. mitis Uo8, and S. pseudopneumoniae RRR472), or S_338_ (S. pneumoniae 14761, S. mitis strains 578 and 661 in cluster X-3). Four PBP2x alleles contained the E_552_ mutation (S. pneumoniae strains F2/F3, URA473, EU14 in cluster X-5, and RRR1136 in X-1). S. pneumoniae strains 5919 (X-3) and F1 contained mutations at both sites. Strains harboring these mutations were nonsusceptible to beta-lactams in cases where their MIC values were known, but varied in MICs between 0.03 (S. pseudopneumoniae RRR472) and >32 μg/ml (S. mitis B6) for penicillin G (mutation at 338), and in the case of mutation E_552_, between 0.12 (S. pneumoniae RR1136) and 0.5 μg/ml (S. pneumoniae EU14) for cefotaxime (Fig. 3; see also Table S2). As cutoff values for resistance were ≤0.25 μg/ml for benzylpenicillin and ≤0.5 μg/ml for cefotaxime as used in a recent study, T_338_ and E_552_ were reported to occur in penicillin-sensitive strains (25).

An indication for the importance of these mutations is the fact that they are occasionally located on a very small mosaic block, for example, in S. pneumoniae RRR1136 (Glu_552_) and SP00058 (Ala_338_), suggesting a selective advantage. The sequence block in S. pneumoniae RRR1136 (codons 550 to 578) was almost identical to the corresponding regions in S. pneumoniae EU14 and S. oralis JH, all encoding E_552_, indicating a common source.

The codon usage of the mutations varied depending on the *pbp2x* allele and the nature of the mosaic block where the mutation occurred (see Table S3). The codon for T_338_ is ACT in S. pneumoniae R6 PBP2x, whereas it is ACC in S. mitis strains M3 and 658. All mutations at this site were caused by a single nucleotide (nt) change except for the double mutation G_338_ in M3-related sequence blocks (ACC to GGC). Similarly, the mutation Q_552_ was caused by a change from CAG or CAA (Q) to GAG/GAA.

### Other mutations associated with penicillin resistance.

Eight mutations were found in both PBP2x classes: V_369_, T_371_, G_378_, L_389_, S_434_, A_513_, I_523_, and Q_531_. Site 597 was changed to S_597_ (S. mitis RRR720) or D_597_ (S. mitis 277) in class 2, whereas it was T_597_ in the class 1 PBP2x of S. mitis B6. D_597_ also occurs in cefotaxime-resistant laboratory mutants (33, 34).

Strikingly, particular combinations of altered amino acids were observed, and some were specifically associated with one cluster (Table S2). This is especially evident in the X-2b cluster, where the mutations at aa 338 (T_338_ or G_338_), F_364_, T_371_, K_417_, S_444_, T_510_, and N_513_ were present in all cases, and T_510_ and N_513_ occurred only in this cluster. These mutations were reported to be associated with resistance by many studies (25, 31, 35–39), which is not astounding, given the fact that the 23F family of PBP2x is common and distributed worldwide among S. pneumoniae and SMG (22). L_389_ and Q_531_ were associated only with PBP2x_M3_-related mosaic blocks that included strains in clusters X-2a, X-2b, and X-4. In cluster X-5, only E_552_ and N_568_ were noticeable, and one strain (S. pneumoniae EU14) contained Q_514_ as well, a site not affected in any other PBP2x. T_371_ plus K_531_ were associated with S_338_ (cluster X-5, S. mitis strains 578, 661, and S. pneumoniae 14761) or P_338_ (S. mitis Uo8 and S. pseudopneumoniae RRR472 in cluster X-3b), but only in one case with A_338_ (S. mitis 315, cluster X-2a). In cluster X-1, A_522_ was altered in most of the S. pseudopneumoniae PBP2x.

Noteworthy is that the mutation N_568_ always occurred in combination with E_552_, including in PBP2x of S. pneumoniae RRR1136 (cluster X-1). L_394_ reported in S. pneumoniae isolates with low-level resistance (38) occurred in several class 2 PBP2x, but never in class 1, i.e., always without changes at sites 338 or 552. Sites that are only altered in strains with high-level resistance are 378 (A_378_ in S. pseudopneumoniae PW2113 and S. pneumoniae SV18 and G_378_ in S. mitis B6), sites 595 and 599 (L_595_/W_599_ in S. mitis B6 and F_595_/H_599_ in S. mitis Uo8), and 336 (M_336_ in S. mitis strains B6 and Uo8). F_595_ has been reported to be associated with high-level resistance to cefotaxime in S. pneumoniae (37).

Most of the mutations mentioned above are positioned around the enzymatic active site and in the 360 to 394 loop, which affects the stability of this region (30) (see Fig. S3). Surface-orientated mutations include those at positions 322, 444, 493 to 495, 507 to 514, 523, and 531. Interestingly, mutations at sites 417, 424, and 434 are directed toward the noncovalent second beta-lactam located between the transpeptidase and the C-terminal domain (40) (Fig. S3).

## DISCUSSION

### Phylogeny of S. pseudopneumoniae.

The identification of viridans streptococci species is notoriously difficult due to their genetic competence, resulting in multiple genomic rearrangements and large accessory genomes. Consequently, physiological tests to define species in this group of bacteria remain unsatisfactory, and therefore current approaches to characterize a large number of strains are mainly based on the comparison of DNA sequences derived from housekeeping genes, i.e., by MLST (7) and MLSA (8).

The MLST-based analysis of 41 atypical S. pneumoniae strains and 39 S. pseudopneumoniae genome sequences (strain IS7394 and 38 whole-genome shotgun sequences) defined a total of 35 (atypical collection) plus 10 strains (genomic data) as S. pseudopneumoniae. MLSA of 110 atypical pneumococci obtained from invasive and noninvasive infections in Spain identified 61 strains as S. pseudopneumoniae, and the authors reported on variable phenotypes among these isolates (19). Similar results have been obtained previously using MLST or MLSA with atypical S. pneumoniae. Forty nontypeable pneumococci from Finland formed a phylogenetic cluster distinct from serotypeable S. pneumoniae (41). Leegaard et al. described 12 atypical pneumococcal isolates from HIV-positive patients (42). Only one of these strains clustered with the type strain S. pseudopneumoniae ATCC BAA-960, which could not clearly be distinguished from S. mitis by MLSA (8), one represented a truly nontypeable S. pneumoniae, and the remaining 10 strains formed a subgroup distinct from S. mitis and S. pneumoniae. Some of the S. pseudopneumoniae strains clustered distinctly from the major group, similar to the four strains in this study, PW2113, PS886, CAP245, and PS3719 (see Fig. 1).

Some shortcomings of the MLST analysis became apparent in the single locus analysis (see Fig. S2 in the supplemental material). Only *aroE* and *recP* sequences placed the majority of the S. pseudopneumoniae strains into one cluster, whereas several smaller clusters were observed with *gki*, *gdh*, and *spi*. A detailed genomic comparison of a significant number of strains will certainly help to identify more genetic determinants specific for the S. pseudopneumoniae core genome for defining the genetic relationship between the atypical strains that clustered outside and within the main S. pseudopneumoniae cluster (Fig. 1). Similarly, features that distinguish members of the different S. mitis and S. oralis branches remain to be clarified. The *gdh* and *spi* MLST alleles singled out a group of S. mitis strains (see Fig. S2D and S2E). MLST placed the recently described strains S. tigurinus AZ-3a (43) and S. dentisani 7747 (44) in the IgA protease-negative S. oralis and the previous S. mitis biovar 2 subcluster, respectively (10), and genomic comparison suggested three subclusters, namely, S. oralis subsp. oralis subsp. nov., S. oralis subsp. tigurinus comb. nov., and S. oralis subsp. dentisani comb. nov., within the coherent phylogenetic clade S. oralis (45). Most important is the strain collection used for phylogenetic analysis, which ideally should consist of randomly collected isolates from different parts of the world. Isolates were frequently only collected from diseased patients, from one hospital, from one particular geographic area, or from a study concentrated on antibiotic-resistant isolates. Given the rapidly growing number of genomes, this shortcoming will be overcome in the near future.

### PBP2x in S. pseudopneumoniae and close relatives.

The aims of this study were to see whether PBP genes of penicillin-sensitive S. pseudopneumoniae are highly conserved, as is the case with S. pneumoniae, and whether they are distinct to alleles identified in truly penicillin-sensitive viridans streptococci. The PBP2x genes in 12 S. pseudopneumoniae strains from Germany, Russia, Denmark, Canada, and Spain were very similar to *pbp2x* in S. pneumoniae R6 but interspersed with small diverse sequence blocks of unknown origin, frequently between codons 246 to 268 and 308 to 329. MIC values of PW2108, CAP156, CAP160, and SK674 were identical to that of S. pneumoniae R6 (MIC values for the other strains within cluster X-1 were not available). Moreover, none of the *pbp2x* alleles in penicillin-sensitive S. pseudopneumoniae were found among S. pneumoniae or S. mitis genome sequences. Therefore, the mosaic makeup of *pbp2x* could serve as a marker for penicillin-sensitive S. pseudopneumoniae. Complex mosaic *pbp2x* sequences were found only in S. pseudopneumoniae isolates with MIC values above those of S. pneumoniae R6, structures which are also typical for penicillin-resistant S. pneumoniae. Three penicillin-resistant strains, IS7394, PW2113, and RRR468, contained the typical sequence block of the widespread 23F family of PBP2x genes (cluster X-2b).

The PBP2x genes in penicillin-sensitive S. pseudopneumoniae strains differed from that in the S. pneumoniae R6 by 35 to 65 nt (1.8 to 2.5%) or 9 to 16 aa, and from each other by up to 23 aa (3.1%) or 120 nt (5.3%). This is clearly above the diversity of *pbp2x* found among penicillin-susceptible S. pneumoniae isolates, which is almost completely conserved. We found a maximum of 12 nt encoding 2 aa differences in *pbp2x* between the R6 strain and S. pneumoniae INV104 scattered throughout the gene. One exception appears to be in *pbp2x* in S. pneumoniae 645.35L98 (29) in that it is identical to *pbp2x* in S. mitis M3, but no genomic information was available and the species identification has not been verified.

On the other hand, the variability of *pbp2x* sequences in penicillin-sensitive S. mitis is astounding, where variation between the individual alleles ranged between 16 and 22%, and a similar phenomenon was observed in S. infantis and S. oralis, where three and five unique alleles and variants, respectively, were detected (Fig. 4). What is the reason for this apparent reduction of *pbp2x* variation from S. mitis to S. pseudopneumoniae and further to S. pneumoniae in the gene encoding a major peptidoglycan synthesizing enzyme? There is no indication that the various *pbp2x* alleles in S. mitis specify subgroups of this species according to the MLST data, and the presence of diverse mosaic genes within sensitive strains suggests that these variations do not result solely from selection pressure imposed by beta-lactam treatment. Since PBP2x is part of a complex protein machinery required for septum synthesis, and thus for cell division (for a review, see reference 46), it would be interesting to study the diversity of genes encoding other cell wall components. Such considerations add to the problem of defining mutations in mosaic PBP2x genes that are associated with resistance as discussed below. We did not consider mutations in the N- or C-terminal domains, as it is generally assumed that only mutations within the transpeptidase domain are important for the expression of resistance. However, they might affect structural features associated with other functions of the proteins or interactions with other partners.

As shown in Fig. 3, the borders of the divergent sequence blocks frequently coincided with the domain structure of PBP2x, i.e., they covered the regions encoding the N- and C-terminal domains and the spacer regions flanking the central transpeptidase domain. Similar observations were obtained with S. pneumoniae R6 transformants obtained with heterologous *pbp2x* (23). Recombination of small blocks has also been observed by Jensen et al. (25) in S. pneumoniae clones (termed microrecombination) (47) and in S. pneumoniae transformants obtained with S. mitis and S. oralis DNA (48, 49). These data support the conclusion that it is not possible to deduce the number of gene transfer events that contribute to a mosaic gene structure.

The mosaic makeup of *pbp2x*, especially in penicillin-resistant strains, depends on the strain collection. For example, *pbp2x* in S. pneumoniae strains from Japan, SP00058, SP00059, and SP00087, display a highly complex mosaic structure. It would be interesting to analyze *pbp2x* sequences in commensal streptococci from Japan to see whether they are related to the three pneumococcal sequences and represent novel alleles. S. pneumoniae appears to be the most promiscuous species, with genes containing sequence blocks of S. mitis, S. oralis, and S. infantis. The *pbp2x* 23F family predominates among resistant strains. In fact, this cluster (X-2b) is the only one that includes all five species investigated in the study, confirming the successful global spread of this gene family. No S. mitis alleles apart from S. mitis M3-related sequences were detected in S. infantis or S. oralis in the small number of alleles investigated here.

### PBP2x mutations associated with penicillin resistance.

There have been several approaches for identifying mutations associated with beta-lactam resistance in S. pneumoniae, but, although they resulted in important conclusions, every single study has drawbacks. The mutations identified in beta-lactam-resistant mutants in the laboratory differed from those in clinical isolates (33, 34). On the other hand, a mutation at 338, which can be selected with oxacillin (32, 33), is one of the few that are clearly associated with resistance in many clinical isolates, but is not changed in laboratory mutants. The most important conclusion was that different pathways for modulating PBP2x exist, and that the mutational pattern is related to the selective beta-lactam. A comparison of the structures of PBP2x derived from the clinical isolates from the 23F family (S. pneumoniae 328 and 5204) (30) and a PBP2x identical with S. pneumoniae F2 (cluster X-5) (50) confirmed this notion.

It is important to note that the two mutations near conserved motifs and linked to resistance by experimental and structural evidence, T/G/P_338_ and E_552_, mediate only a 1.5-fold increase in the resistance level for penicillin, which is 0.03 μg/ml in the penicillin-sensitive strain S. pneumoniae R6 and up to 2.25-fold for cefotaxime (MIC, 0.02 μg/ml) depending on the mutation (32, 33, 36, 51). Not surprisingly, the MICs of strains carrying these mutations varied largely, clearly implicating other sites relevant for the resistance phenotype. Thus, it is impossible to directly correlate a mutation in PBP2x and the MIC of a resistant clinical isolate.

**FIG 5:**
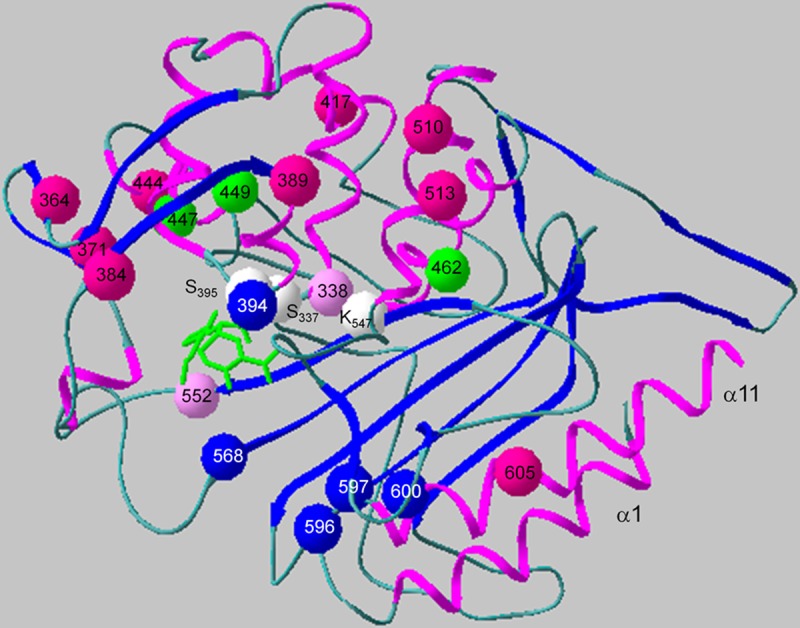
Position of mutations in PBP2x implicated in beta-lactam resistance. Shown is the structure of the transpeptidase domain of the acylated form of PBP2x in S. pneumoniae R6 with cefuroxime (1QMF.pdb; [30]). The positions of mutations mentioned in the Discussion are indicated. Light pink, mutations near active site residues; pink, signature mutations of the 23F family with G_384_ being present in the penicillin-sensitive reference strain S. mitis M3; blue, mutations in other PBP2x families; green, signature residues of the X-5 family present also in penicillin-sensitive strains; white, active-site residues. The cefuroxime molecule in the active site is shown in green.

Depending on the strain collection, the mutations identified as being associated with penicillin resistance vary considerably, since different clones, and thus, the frequency of the various mosaic structures of PBP2x, prevail in different geographic areas. PBP2x of the 23F type is common among penicillin-resistant S. pneumoniae, S. mitis, S. infantis, and S. oralis (22), and as shown here, also in S. pseudopneumoniae. Thus it is not surprising that amino acid changes associated with this PBP2x family (F_364_, T_371_, L_389_, K_417_, S_444_, T_510_, N_513_, and T_605_; Fig. 5) are mentioned in many studies (23, 25, 35, 37, 38, 52, 53). However, other mutations listed in these studies frequently included alterations that occur in the penicillin-sensitive reference strains of this study. Two crystal structures of PBP2x of the 23F family are available, namely, for S. pneumoniae strain 328 (30) and strain 5204 (35). It was necessary to mutate F_364_ back to leucine to prevent proteolysis of the protein in the PBP2x_328_, an indication that this site plays a critical role in the structure of PBP2x. *In vitro* mutagenesis of *pbp2x*_5204_ combined with biochemical and microbiological tests singled out six mutations critical for resistance: T_371_, A_338_, F_339_, G_384_, T_400_, and T_605_ (35). However, only T_371_, A_338_, and T_605_ are common mutations, and G_384_ is frequent in PBP2x variants of penicillin-susceptible Streptococcus sp. (Fig. 3; see also Table S2). When these six mutations were introduced into S. pneumoniae R6, the PBP2x protein differed by an order of magnitude in acylation efficiency compared with that of the 5204-PBP2x, suggesting a cooperative role of some other substitutions (35). Cooperativity of alterations might explain why the reversal of G_384_, common in penicillin-susceptible strains, decreased benzylpenicillin and cefotaxime MICs in a S. pneumoniae with high-level resistance (37).

Most importantly, the mutational makeup of mosaic PBP2x variants differed between the PBP2x groups. This supports the notion of distinct mutational pathways for PBP2x, but puts this conclusion in the context of the parental PBP2x variants of sensitive strains. Given the fact that the amino acid sequences of PBP2x in penicillin-susceptible S. mitis vary by up to >22%, it is conceivable that certain mutations are allowed only in the particular structural context of individual PBP2x. For example, A_338_ was mutated to S_338_ or P_338_ in cluster X-3, whereas mutations A_338_ or G_338_ were associated with X-2a/2b, and E_552_ was frequent in cluster X-5. A comparison of structural data available from a PBP2x identical to that of strain S. pneumoniae F2 (cluster X-5) (50) with PBP2x of the 23F family (30, 35) fully supports alternative mechanisms of penicillin resistance.

N_568_, which always occurred in combination with E_552_, is also an active-site mutation with its side chain lying across from that of E_552_ as deduced from the PBP2x structure identical to PBP2x_F2_ (X-5) (50). The mutations K_417_, S_424_, and S_434_ are located between the between the transpeptidase and the C-terminal domains near the second noncovalent cefuroxime molecule (40), similar to mutations in laboratory mutants (54), supporting the importance of the C-terminal domain for antibiotic and, most likely, substrate recognition. Some mutations proposed to play a role in resistance, and which also have structural effects on the active site, are present in sensitive S. mitis, including M_447_ and A_449_, signature residues for PBP2x of cluster X-5 (Fig. 4), and G_384_. Such changes must affect the interaction with the actual substrate as well, suggesting different enzymatic properties of PBP2x variants already in penicillin-susceptible streptococci.

In conclusion, penicillin-sensitive S. pseudopneumoniae strains contain particular *pbp2x* alleles distinct from those of the close relatives, S. pneumoniae and S. mitis. PBP2x genes in resistant S. pseudopneumoniae display complex mosaic structures that are typical for resistant strains of other viridans streptococci. PBP2x sequences of four penicillin-sensitive S. mitis isolates account for most of the diverse sequence blocks in resistant S. pneumoniae and S. mitis, and are widespread among sensitive S. mitis as well. Altered sites associated with resistant strains revealed specific patterns depending on the parental sequence block, in agreement with distinct mutational pathways during the development of beta-lactam resistance. Several sites have been shown to affect acylation kinetics to beta-lactam antibiotics, and it is conceivable that the proteins display slightly different enzymatic properties with their actual substrate, which still needs to be characterized. Characterization of the mosaic structure of PBPs in penicillin-resistant isolates by comparison to parental genes of sensitive strains is not only helpful but probably essential for identifying mutations related to resistance, which needs to be evaluated by further genetic, biochemical, and structural data. Given this scenario, it is unlikely that any novel antibiotic will target all PBP2x variants equally well, unless by a mechanism completely distinct from that of beta-lactams.

## MATERIALS AND METHODS

### Characterization of bacterial strains and antibiotic susceptibility testing.

Forty-one strains isolated between 1997 and 2011 from the German National Reference Center for Streptococci (GNRCS) were included in the present study (see Table S1A in the supplemental material). They were characterized as atypical S. pneumoniae by serotyping and optochin and bile (deoxycholate) susceptibility, and were tested for the presence of the capsular biosynthesis gene *cpsA*.

Pneumococcal isolates were serotyped by the Neufeld Quellung reaction using type and factor sera provided by the Statens Serum Institute, Copenhagen, Denmark. LytA PCR/restriction was performed as described previously (15). PCR amplification of 16S rRNA genes and subsequent cleavage with the restriction endonuclease BsiHKAI was performed according to published procedures (55).

Identification by MLST was performed as described previously (7) (Table S1B). In addition, the species from 38 genomes available in GenBank and listed under “whole-genome contigs/S. pseudopneumoniae” were characterized by *map* and *pyk*, which differentiate S. pseudopneumoniae from S. mitis in MLSA (8), and by *pbp3*, which differentiates streptococcal species. Those genomes identified as S. mitis and S. pseudopneumoniae were further characterized by MLST, and details are listed in Table S1B.

All strains were tested for antibiotic MICs using the broth microdilution method as recommended by the CLSI (56). The microtiter plates (Sensititre NLMMCS10; TREK Diagnostic Systems, Ltd., East Grinstead, UK) contained penicillin G (PEN), cefotaxime (CEF), clarithromycin/erythromycin (CLA/ERY), clindamycin (CLI), tetracycline (TET), levofloxacin (LEV), chloramphenicol (CHL), and trimethoprim-sulfamethoxazole (SXT) with cation-adjusted Mueller-Hinton broth (Oxoid, Wesel, Germany) and 5% lysed horse blood. Oxacillin susceptibility was tested using 1-μg discs (Oxoid, Wesel, Germany). In some isolates, MICs for beta-lactams were additionally specified using narrow dilutions of the antibiotic as described previously (23).

### DNA isolation and PCR amplification.

Chromosomal DNAs from streptococci were isolated as described previously (23). PCR products were purified using a JetQuick DNA purification kit (GenoMed). PCRs were performed using either Goldstar Red *Taq* polymerase (Eurogentec) or DreamTaq polymerase (Fermentas) according to the manufacturer's instructions. The oligonucleotides used in this study were obtained from Eurofins. PBP2x gene fragments were amplified with the primers pn2xup and pn2xdown (23), and direct sequencing of PCR products was performed with consecutive primers.

### Bioinformatic tools and analysis.

Neighbor-joining trees were generated with MEGA6.06 (57) and Clustal alignments using standard parameters. Bootstrap analysis was based on 1,000 replicates. PBP2x sequences were aligned by ClustalX2 (58) and further processed by Genedoc (http://www.psc.edu/biomed/genedoc). PBP2x gene sequences were aligned with the each of the reference sequences. Codon sites were included manually and trimmed by the program Clustal Formatter (http://nbc11.biologie.uni-kl.de/) to reveal only sites that differ from the reference sequence as shown in Table S2. Sequence blocks that differ by <5% are defined as distinct sequences as shown by different colors in Fig. 3 and 4.

### Accession number(s).

Accession numbers of *pbp2x* sequences are KY292535, KY292538, KY292539, KY292542, KY292549 to KY292551, KY292559, KY292560, KY292564, AJ238585, KY292528, KY292530, KY292531, KY292533, KY292534, KY292536, KY292540, KY292541, KY292543, KY292544, KY292548, KY292552 to KY292557, and KY292561 to KY292563 and are listed in Table S2.

## Supplementary Material

Supplemental material
