# Supplementary material for: Insight into the Diversity of Penicillin-Binding Protein 2x Alleles and Mutations in Viridans Streptococci
Source: Antimicrob Agents Chemother. 2017 Apr 24;61(5):e02646-16. doi: 10.1128/AAC.02646-16 (PMC5404556; doi:10.1128/AAC.02646-16)
Supplement: Supplemental material [file supp_61_5_e02646-16__index.html]

Insight into the Diversity of Penicillin-Binding Protein 2x Alleles and Mutations in Viridans Streptococci — Supplemental material 

# Insight into the Diversity of Penicillin-Binding Protein 2x Alleles and Mutations in Viridans Streptococci

## Supplemental material

- Supplemental file 1 -

  DATASET S1: Table S1

  XLSX, 29K
- Supplemental file 2 -

  DATASET S2: Table S2

  XLSX, 137K
- Supplemental file 3 -

  DATASET S3: Table S3

  XLSX, 12K
- Supplemental file 4 -

  TEXT S1: Fig. S1, Fig. S2, Fig. S3

  PDF, 1.2M
