# Supplementary material for: Insight into the Diversity of Penicillin-Binding Protein 2x Alleles and Mutations in Viridans Streptococci
Source: Antimicrob Agents Chemother. 2017 Apr 24;61(5):e02646-16. doi: 10.1128/AAC.02646-16 (PMC5404556; doi:10.1128/AAC.02646-16)
Supplement: Supplemental material [file AAC.02646-16_zac004176086s4.pdf]

Supplementary Figures

Figure S1.
Phylogenetic analysis of the PBP3 gene from *S. pseudopneumoniae* genomes. PBP3 sequences were extracted from *S. pseudopneumoniae* whole-shotgun genome sequences (red triangles) and compared with reference genomes of *S. pneumoniae* (strains R6 and ATCC 700669), *S. pseudopneumoniae* strain IS7394 (1), *S. mitis* (strains SK1080, accession No. AFQV01000021.1; B6 (2) and NCTC12261, accession No. PRJNA173, *S. infantis* DD18 (3) and *S. oralis* Uo5 (4). Bootstrap values (percentages) are based on 1,000 replications. The bar refers to genetic divergence as calculated by the MEGA software.

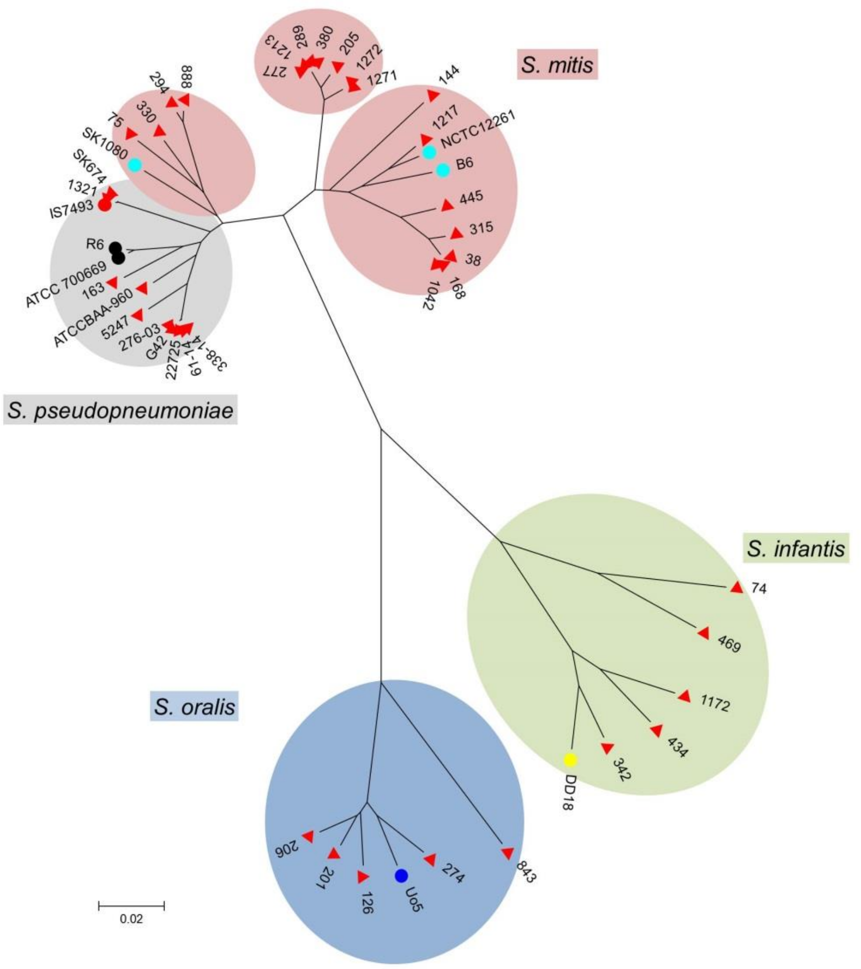

Figure S2.

Neighbor-joining trees for the seven MLST loci. S2A-G: Phylogenetic trees were constructed using individual sequences of each of the seven MLST loci as indicated from all 41 atypical *S. pneumoniae* strains of this study combined with reference *S. mitis* and *S. pneumoniae* from Chi et al. (5). In addition, MLST genes were extracted from the genomes of *S. pseudopneumoniae* IS7394, 9 *S. pseudopneumoniae* and 19 genomes from GenBank listed under whole-genome shotgun contigs of *S. pseudopneumoniae* (Table S1b). *S. oralis* Uo5 was included as reference for this species. Strains from Chi *et al*. are shown by colors indicating the origin of the strains. Grey areas indicate clusters of *S. pseudopneumoniae*. Bootstrap values (percentages) are based on 1,000 replications. The bar refers to genetic divergence as calculated by the MEGA software.

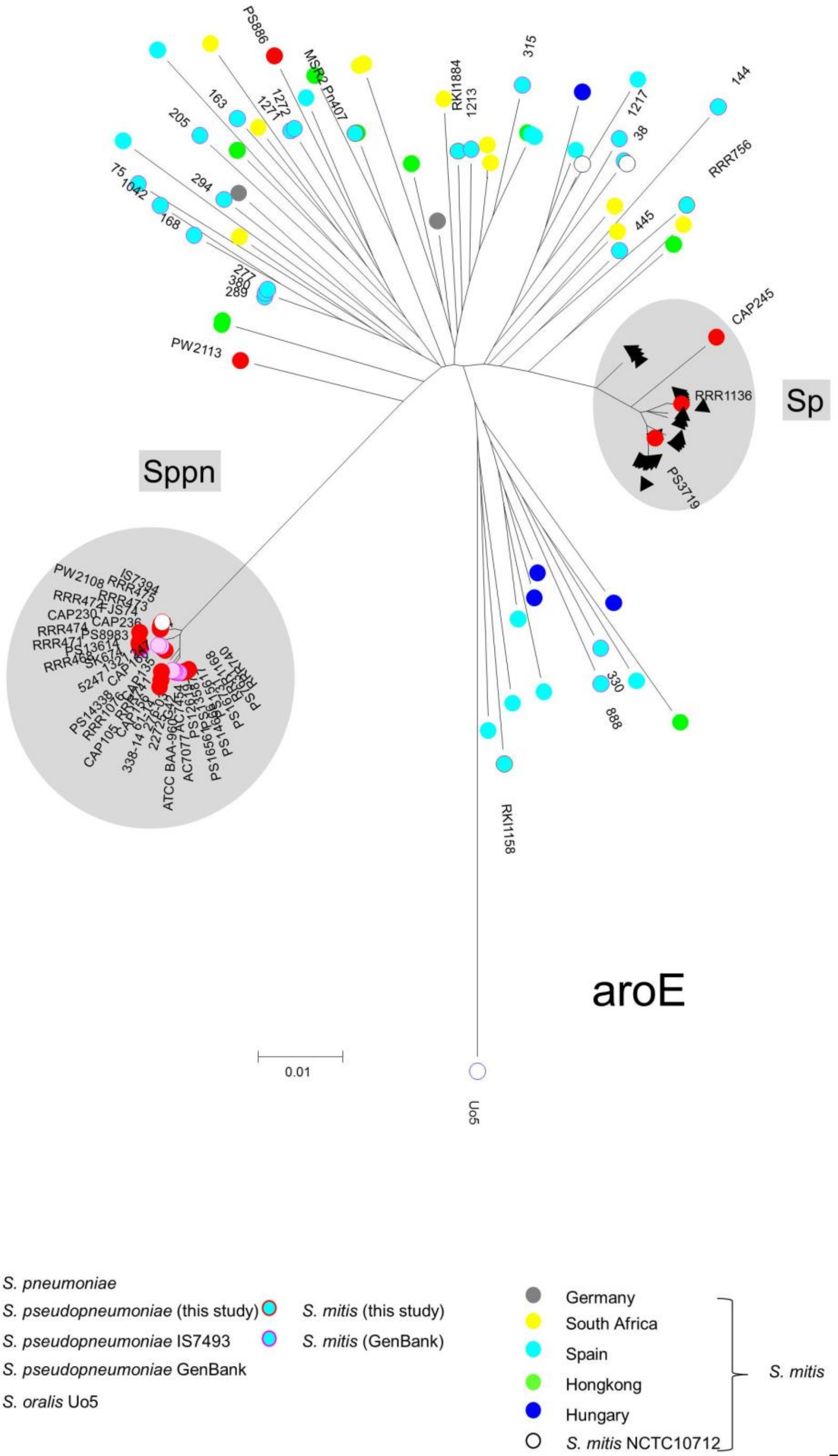
aroE
Sppn
Sp
0.01
PS886
MSP2 Pn407
RKI1884
1213
315
144
1217
38
RRR756
445
163
1272
1271
205
294
75
1042
168
277
380
289
PW2113
CAP245
RRR1136
PS3719
330
888
RKI1158
Uo5
S. pneumoniae
S. pseudopneumoniae (this study)
S. pseudopneumoniae IS7493
S. pseudopneumoniae GenBank
S. oralis Uo5
S. mitis (this study)
S. mitis (GenBank)
Germany
South Africa
Spain
Hongkong
Hungary
S. mitis NCTC10712
S. mitis

Figure S2A

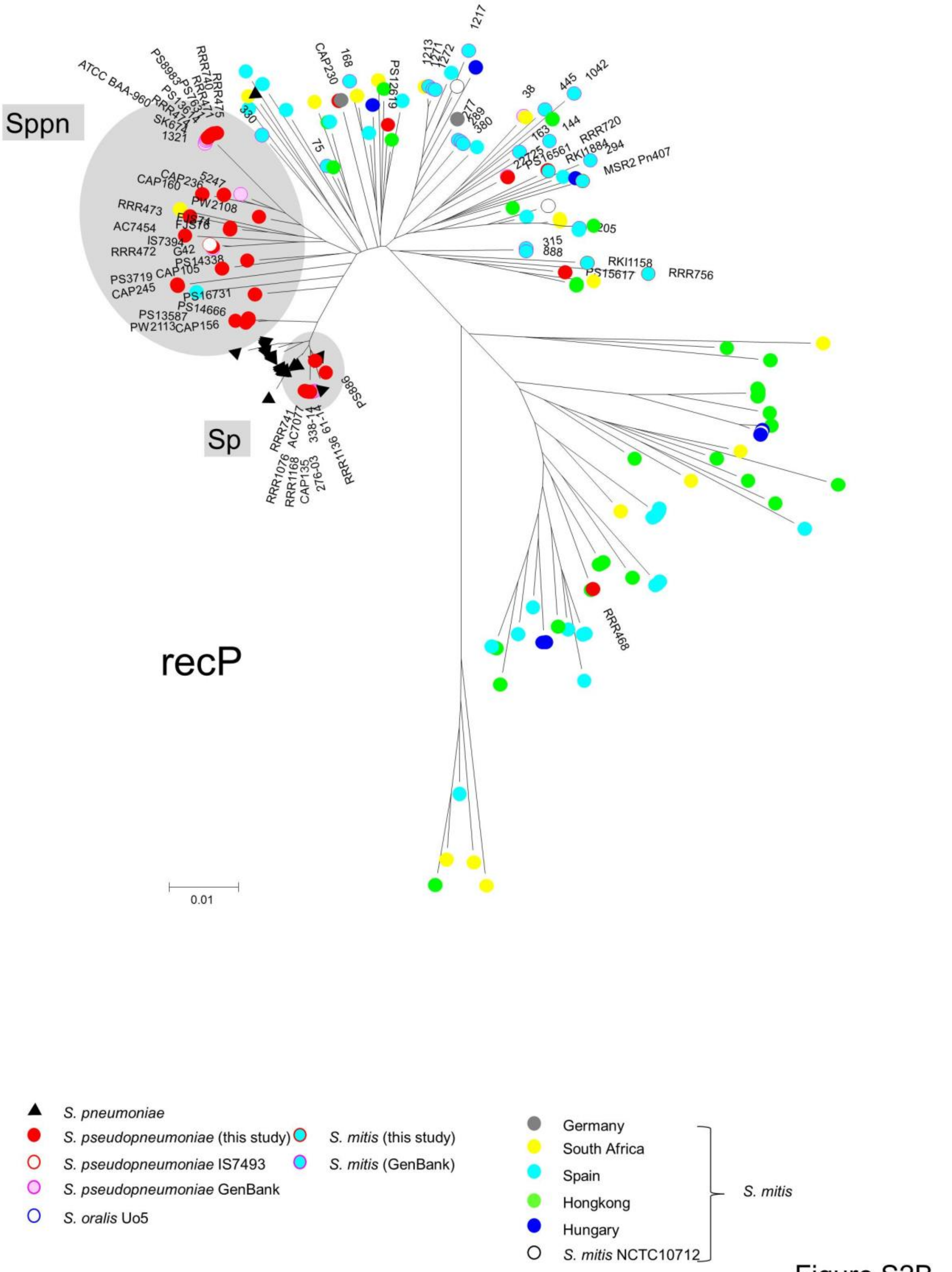

Sppn
Sp
recP
0.01
S. pneumoniae
S. pseudopneumoniae (this study)
S. pseudopneumoniae IS7493
S. pseudopneumoniae GenBank
S. oralis Uo5
S. mitis (this study)
S. mitis (GenBank)
Germany
South Africa
Spain
Hongkong
Hungary
S. mitis NCTC10712
S. mitis

Figure S2B

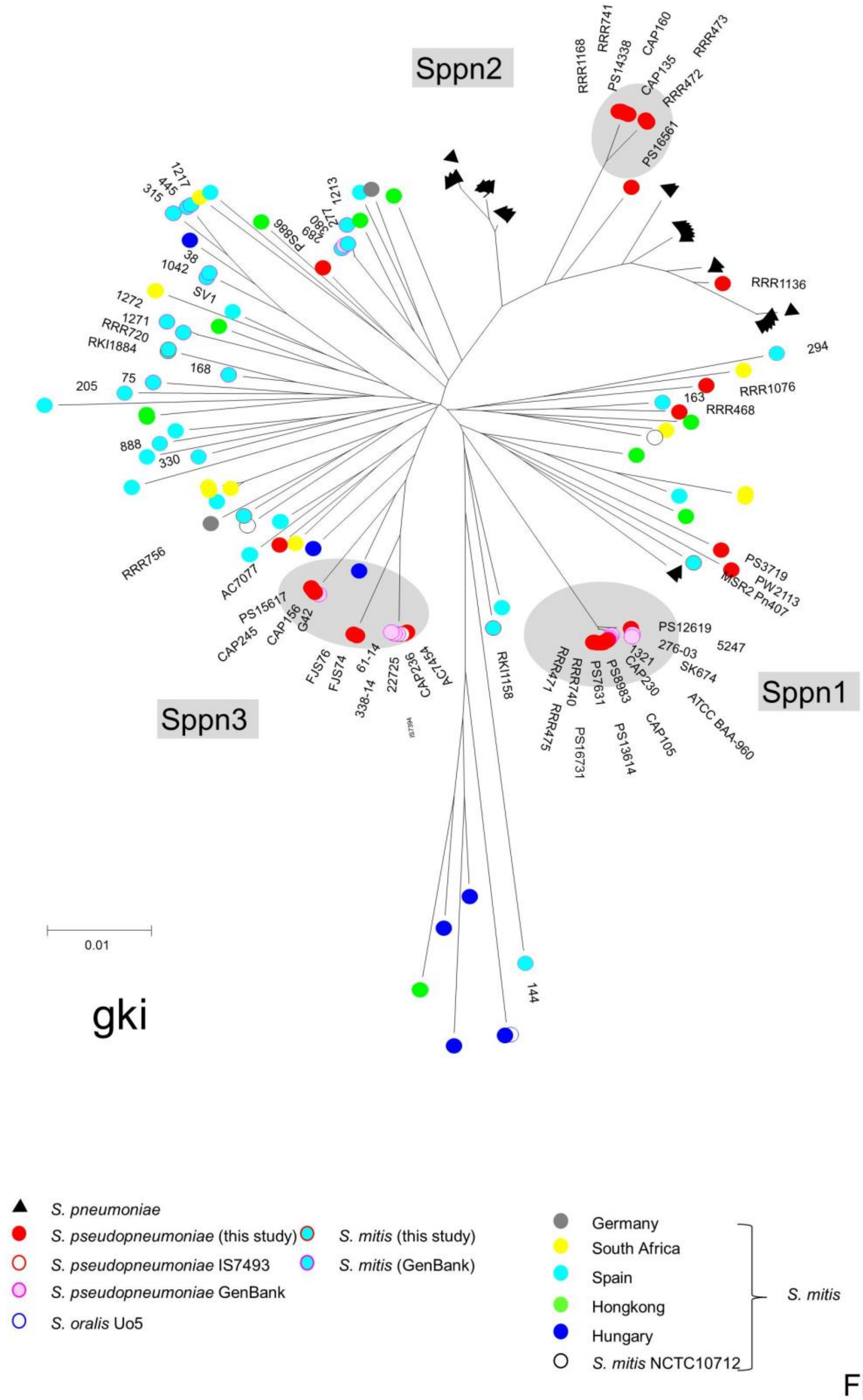

Sppn2
Sppn1
Sppn3
RRR1168
RRR741
PS14338
CAP160
RRR473
CAP135
RRR472
PS16561
RRR1136
294
RRR1076
163
RRR468
PS3719
PW2113
MSR2 Pn407
PS12619
5247
276-03
SK674
1321
CAP230
PS8983
PS7631
RRR740
RRR471
RRR475
PS16731
PS13614
CAP105
ATCC BAA-960
RKI1158
144
AC7454
CAP236
22725
61-14
338-14
FJS74
FJS76
G42
CAP156
CAP245
PS15617
AC7077
RRR756
888
330
205
75
168
RKI1884
RRR720
1271
1272
SV1
1042
38
315
445
1217
PS886
269
2080
277
1213
0.01
gki
S. pneumoniae
S. pseudopneumoniae (this study)
S. pseudopneumoniae IS7493
S. pseudopneumoniae GenBank
S. oralis Uo5
S. mitis (this study)
S. mitis (GenBank)
Germany
South Africa
Spain
Hongkong
Hungary
S. mitis NCTC10712
S. mitis

Figure S2C

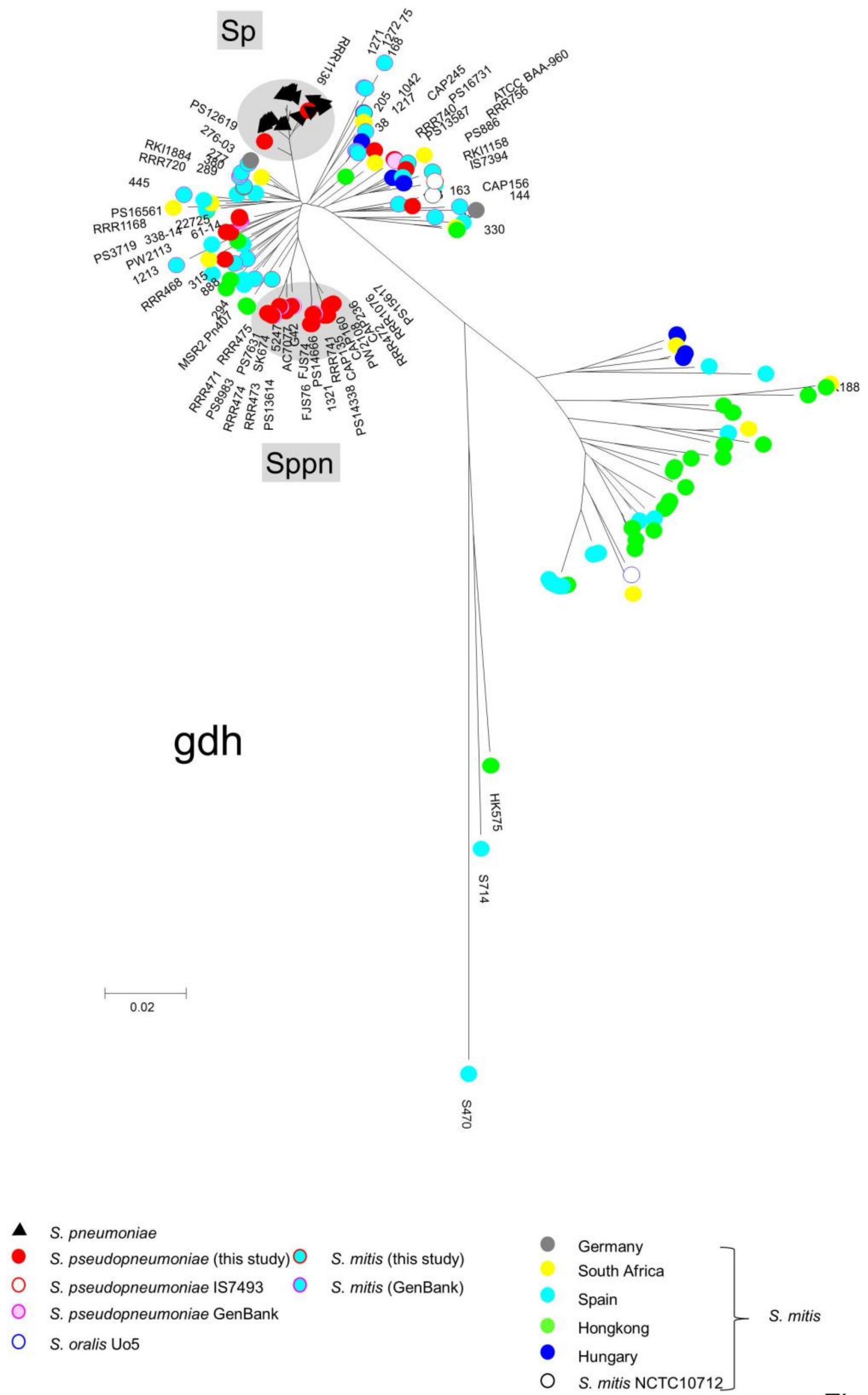
Sp
Sppn
gdh
0.02
S. pneumoniae
S. pseudopneumoniae (this study)
S. pseudopneumoniae IS7493
S. pseudopneumoniae GenBank
S. oralis Uo5
S. mitis (this study)
S. mitis (GenBank)
Germany
South Africa
Spain
Hongkong
Hungary
S. mitis NCTC10712
S. mitis

Figure S2D

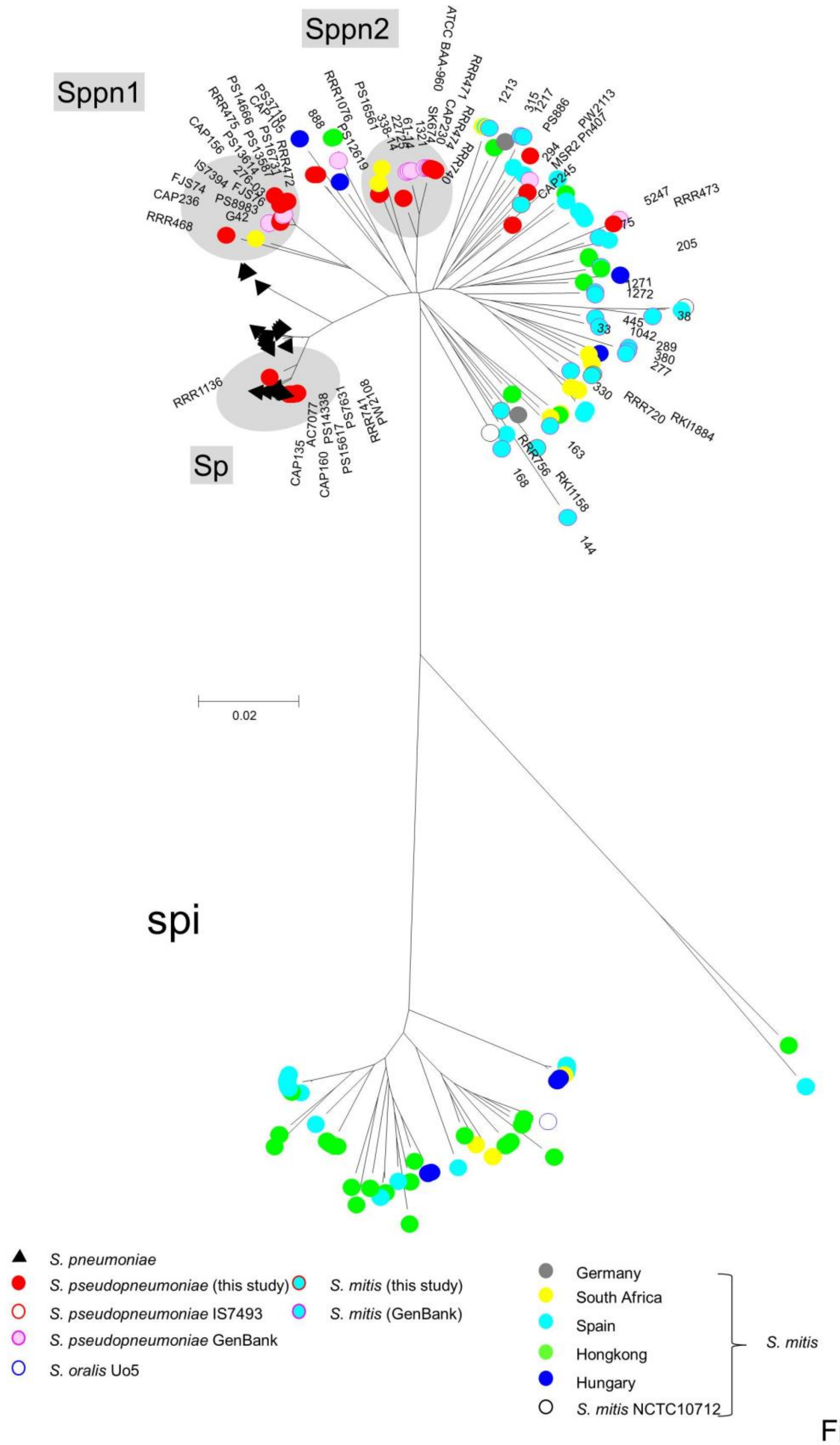
Sppn1
Sppn2
Sp
spi
0.02
ATCC BAA-960
RRR468
G42
CAP236
FJS74
PS8983
IS7394
RRR1136
CAP135
AC7077
CAP160
PS14338
PS15617
PS7631
RRR741
PW2108
888
RRR1076
PS16561
PS12619
SK674
CAP230
RRR740
RRR471
RRR474
1213
315
1217
PS886
PW2113
Pn407
MSR2
294
CAP245
5247
RRR473
205
1271
1272
38
445
1042
33
289
380
277
330
RRR720
RKI1884
163
RRR756
RKI1158
168
144
S. pneumoniae
S. pseudopneumoniae (this study)
S. pseudopneumoniae IS7493
S. pseudopneumoniae GenBank
S. oralis Uo5
S. mitis (this study)
S. mitis (GenBank)
Germany
South Africa
Spain
Hongkong
Hungary
S. mitis NCTC10712
S. mitis

Figure S2E

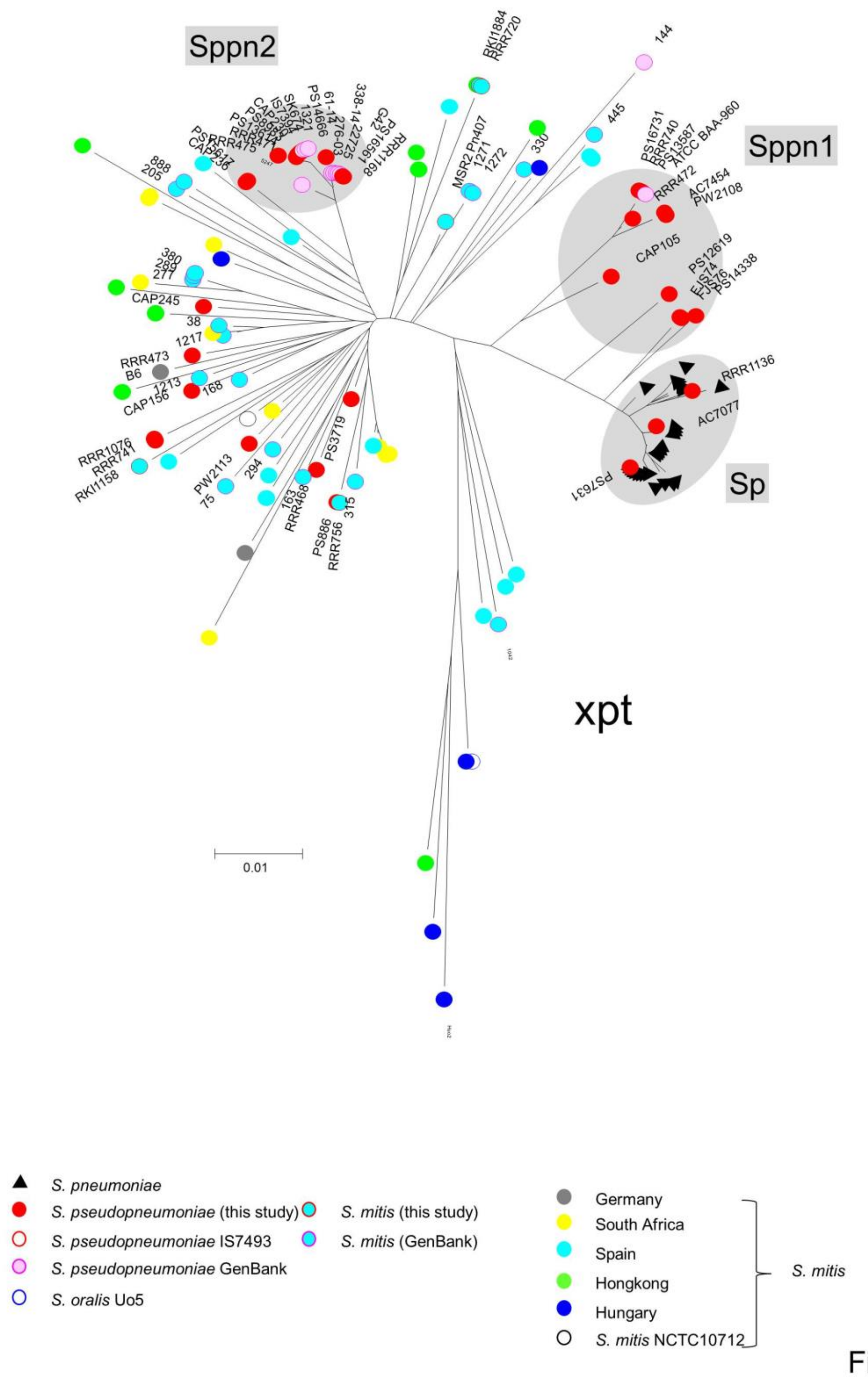
Sppn2
Sppn1
Sp
xpt
0.01
RKI1884
RRR720
144
445
PS16731
RRR740
PS13587
ATCC BAA-960
RRR472
AC7454
PW2108
CAP105
PS12619
PS14338
RRR1136
AC7077
PS7631
MSR2 Pn407
1271
1272
330
338-14
G42
PS16561
RRR1168
PS14666
888
205
380
289
277
CAP245
38
1217
RRR473
B6
1213
168
CAP156
RRR1076
RRR741
RKI1158
PW2113
75
294
PS3719
163
RRR468
PS886
RRR756
315
S. pneumoniae
S. pseudopneumoniae (this study)
S. pseudopneumoniae IS7493
S. pseudopneumoniae GenBank
S. oralis Uo5
S. mitis (this study)
S. mitis (GenBank)
Germany
South Africa
Spain
Hongkong
Hungary
S. mitis NCTC10712
S. mitis

Figure S2F

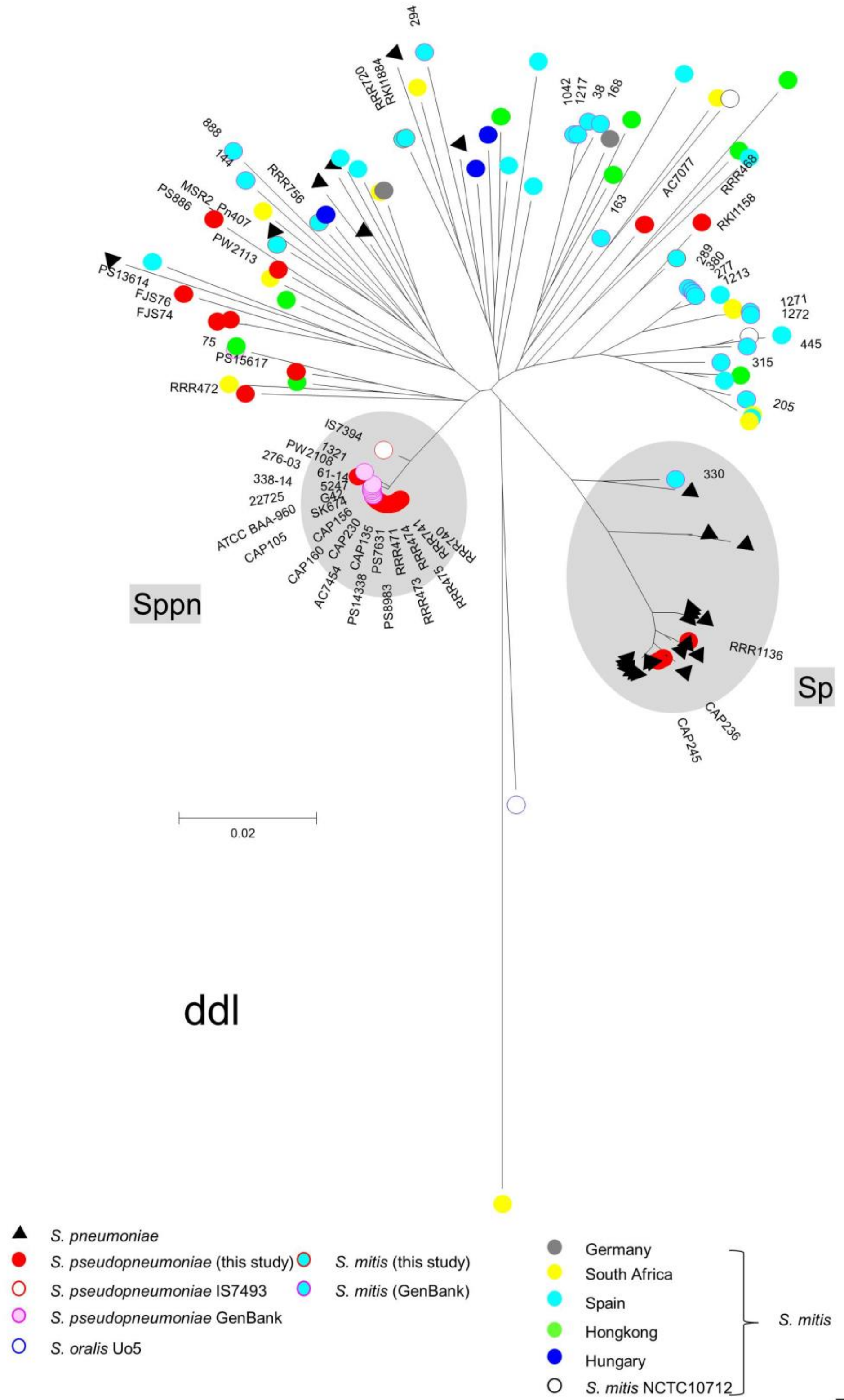
Sppn
Sp
ddl
0.02
S. pneumoniae
S. pseudopneumoniae (this study)
S. pseudopneumoniae IS7493
S. pseudopneumoniae GenBank
S. oralis Uo5
S. mitis (this study)
S. mitis (GenBank)
Germany
South Africa
Spain
Hongkong
Hungary
S. mitis NCTC10712
S. mitis

Figure S2G

Figure S3.
Position of mutations in the transpeptidase domain of PBP2x. Shown is the structure of the transpeptidase and C-terminal domains of the acylated form of PBP2x of *S. pneumoniae* R6 with cefuroxime [1QMF.pdb; (6)]. Cefuroxime molecules are shown in green; white arrow: the non-covalent bound molecule positioned between the two domains.
The positions of mutations highlighted in red in Table S2 are shown. Light pink: mutations close to active site residues; orange: signature mutations of the 23F family; blue: mutations highlighted in the discussion; red: all other mutations. White: active site residues.

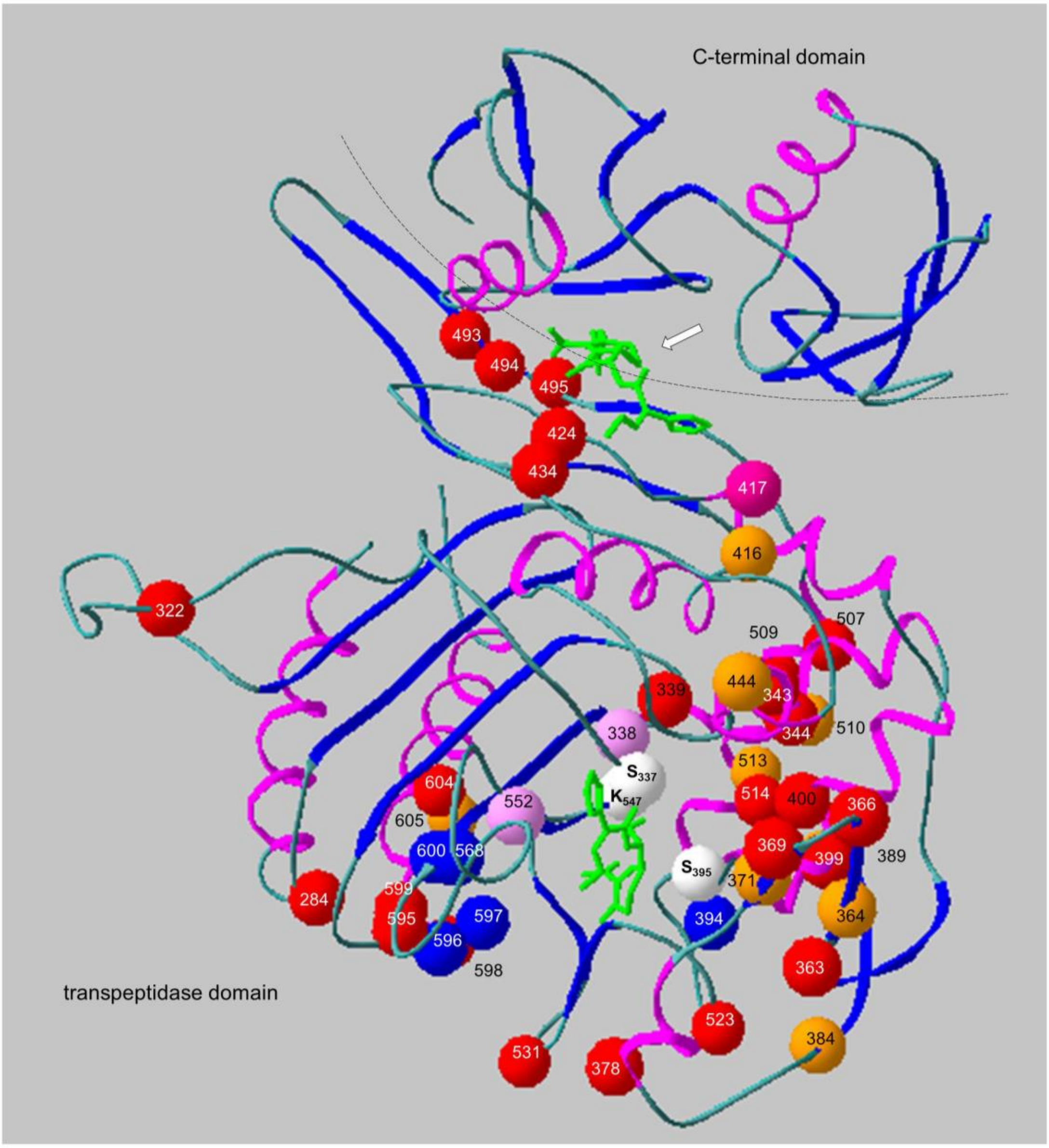

References

1. **Shahinas, D., C. S. Thornton, G. S. Tamber, G. Arya, A. Wong, F. B. Jamieson, J. H. Ma, D. C. Alexander, D. E. Low, and D. R. Pillai**. 2013. Comparative genomic analyses of *Streptococcus pseudopneumoniae* provide insight into virulence and commensalism dynamics. PLoS. ONE. **8**:e65670. doi:10.1371/journal.pone.0065670 [doi];PONE-D-13-07093 [pii].
2. **Denapaite, D., R. Brückner, M. Nuhn, P. Reichmann, B. Henrich, P. Maurer, Y. Schähle, P. Selbmann, W. Zimmermann, R. Wambutt, and R. Hakenbeck**. 2010. The genome of *Streptococcus mitis* B6 - what is a commensal? PLoS ONE. **5**:e9426.
3. **Denapaite, D., M. Rieger, S. Köndgen, R. Brückner, I. Ochigava, P. Kappeler, K. Mätz-Rensing, F. Leendertz, and R. Hakenbeck**. 2016. Highly variable *Streptococcus oralis* strains are common among viridans streptococci isolated from primates. mSphere **1**:e00041-15. doi: doi: 10.1128/mSphere.00041-15.
4. **Reichmann, P., M. Nuhn, D. Denapaite, R. Brückner, B. Henrich, P. Maurer, M. Rieger, S. Klages, R. Reinhard, and R. Hakenbeck**. 2011. Genome of *Streptococcus oralis* strain Uo5. J. Bacteriol. **193**:2888-2889.
5. **Chi, F., O. Nolte, C. Bergmann, M. Ip, and R. Hakenbeck**. 2007. Crossing the barrier: evolution and spread of a major class of mosaic *pbp2x* in *S. pneumoniae, S. mitis* and *S. oralis*. Int. J Med. Microbiol. **297**:503-512.
6. **Dessen, A., N. Mouz, E. Gordon, J. Hopkins, and O. Dideberg**. 2001. Crystal structure of PBP2x from a highly penicillin-resistant *Streptococcus pneumoniae* clinical isolate: a mosaic framework containing 83 mutations. J. Biol. Chem. **276**:45105-45112.
